# Workmen's Compensation: Its Medical Aspect

**Published:** 1897-05-08

**Authors:** 


### WORKMEN'S COMPENSATION: ITS MEDICAL ASPECT.

The Bill introduced on Monday night by Sir M. W. Ridley, although confessedly a tentative measure, and one whose operation will not extend over the whole field of industry, is one which will throw on the medical profession a good deal of work of a sort that most medical men would gladly be without.

If there is one part of a doctor's work which is more poorly paid than another, if there is one part more irksome, which involves greater responsibility, which receives less thanks, and, we may add, which involves the exercise of greater skill than another, it is the giving of medical certificates of incapacity for work. A moment's consideration of this Bill will show that on the medical man is thrown the final responsibility of deciding as to a man's capacity or incapacity for the performance of his duties; as indeed must of necessity be the case. To understand how great this responsibility is likely in some cases to be we must read the first schedule, which provides that, "in case of incapacity for work there shall be weekly payments during the incapacity, after the second week, not exceeding 50 per cent. of his earnings at the time of the accident, such payments not to exceed £1."

When this statement was made, Sir Charles Dilke immediately asked, "What results in the case of permanent incapacity?" (Hear, hear.) Sir R. Webster said, "The words are, 'during incapacity,'" and to this Sir M. W. Ridley replied, "That is so. I believe the House will be of opinion that the limit adopted by the Government is a safe and reasonable one."

Now all medical men who have had much to do with the giving of certificates, and especially with such as deal with personal injuries, will see at once what difficult problems will be presented to them for solution under this schedule.

An offer of 50 per cent. of the normal wage so long as incapacity may last opens to the medical eye, on the one hand, a long vista of malingerers seeking for permanent pensions, and on the other a miserable crew of real sufferers from neurasthenia asking for official certification of their subjective wretchedness. In either case it holds out a prospect of many problems extremely difficult of solution, and full of responsibility in consequence of the money loss which will be caused to one side or the other if any mistake be made. Herein lurks a danger which medical men will do well to bear in mind.

From the master and workman's point of view, a somewhat important question is involved in the interpretation which may be attached to the term incapacity for work.

We feel bound to believe that the intention of the Bill is that this shall mean incapacity for continuing the work which the workman was doing up to the time of the accident. If it meant anything else the workman would receive no compensation so long as he could sweep a crossing! Take the case of a railway guard who loses his leg in consequence of an accident. This is a plain-sailing case, and he will apparently receive a pension for life at the rate of 50 per cent. of the wages he was having before the time of the accident. But what is to be done with those whose accidents, while incapacitating them from doing their old work, leaves them fairly able to get along in some other way?

The Friendly Societies are very strict about these cases, and take great care that men receiving benefit shall not eke out their pay by doing odd work—even turning the mangle for their wives; and if the definition of incapacity is to mean only incapacity for the old employment, the State will have to decide whether those who are incapacitated according to the Act are to remain idle for the rest of their lives or are to be allowed to undertake some other work. To condemn them to idleness would in our view be a great cruelty, while on the other hand there can be no doubt that to do otherwise will be a great temptation to a form of—fraud shall we call it? which will be very difficult to deal with. There are scores of men about forty-five years of age who know well that in a few years they will find their work too much for them. When such a man, say an engineer earning a couple of pounds a week, meets with an accident which incapacitates him from work, and sees visions of a pound a week for life before him, we may be sure that he will not be anxious to be declared fit for duty; and the responsibility thrown upon a medical man of declaring him to be so will be a very heavy one, for it must be remembered that, however well a man of that age may apparently recover, he may never be quite the same man again. He is already on the down grade, and every month is likely to make him less likely ever to take up his old position. Under such circumstances it will become a most difficult task for any medical man to decide how much of his deterioration is due to advancing age and how much to the accident that has made the patient conscious of his lessening powers.

We cannot but think then that this Bill should contain some definition of the meaning of the term incapacity for work, and some provision for the establishment of a permanent medical court which should undertake the responsible task of deciding the medical questions which are sure to arise in its administration.

